# Living a successful weight loss after severe obesity

**DOI:** 10.1080/17482631.2018.1487762

**Published:** 2018-06-27

**Authors:** Eli Natvik, Målfrid Råheim, John Roger Andersen, Christian Moltu

**Affiliations:** a Department of Health and Caring Sciences, Western Norway University of Applied Sciences, Førde, Norway; b Center for Health Research, District General Hospital of Førde, Førde, Norway; c Department of Global Public Health and Primary Care, University of Bergen, Bergen, Norway; d Division of Psychiatry, District General Hospital of Førde, Førde, Norway

**Keywords:** Embodiment/bodily experiences, lifestyle change, lived experience, obesity, phenomenology, well-being

## Abstract

**Purpose:** Losing weight and keeping it off for the long term is difficult. Weight regain is common. Experiences of successful non-surgical weight loss after severe obesity are largely unexplored. We know little about long-term weight loss processes, and how health care services can be of help to those living them. **Methods:** Drawing on in-depth interviews of 8 women and 2 men, the aim of this phenomenological study is to describe the experiences of adults who have been severely obese, who have lost weight and maintained weight loss for the long term (>5 years). **Results:** Findings show that after severe obesity, sustained weight loss has no endpoint, yet is always easy to end. Keeping weight off means committing to oneself, continuing profound changes and cultivating sensitivity towards oneself and others. A phenomenological understanding of sustained weight loss can inform professionals who deal with health issues and challenges occurring in the life of people leaving severe obesity.

From being an unusual condition in the 1970s, the prevalence of severe obesity is increasing in several developing countries. If current trends continue, we are facing an epidemic of severe obesity (NCD risk factor collaboration, 2016). Severe obesity is important to attend to, regarding population health and in clinical encounters. Obesity-related issues may threaten vital life functions and limit lifespan. Health risk rises with increasing body weight, and obesity is related to coronary heart disease, hypertension, type 2 diabetes, musculoskeletal disorders, some cancers, depression and anxiety (National Institute for Health and Care Excellence, 2006; National Institutes of Health, 2012; Prospective Studies Collaboration, 2009; World Health Organization, 2017a). Experiences, meanings and attitudes attached to obesity differ profoundly according to severity (Lewis et al., 2010).

Social stigma, discrimination and withdrawal from social life and loved ones are consequences for many (Brewis, 2014; Puhl, 2011; Puhl & Heuer, 2009). Weight stigma is part of clinical encounters, and health care providers are not necessarily reflective about this (Malterud & Ulriksen, 2011; Puhl & Heuer, 2009; Setchell, Gard, Jones, & Watson, 2017). Weight stigma in clinical encounters is a paradox, because stigma affects patients’ health and quality of life negatively, and undermines trust between patients and health care providers (Gudzune, Bennett, Cooper, & Bleich, 2014; Setchell et al., 2017). This means that health care providers and patients encounter a tension between supporting initiatives towards weight loss and change, and the risk of enacting stigma and affecting health negatively.

People’s lived experiences can yield rich examples to help understand long-term weight loss in concrete ways, and providing an in-depth contextual understanding may guide professional action and strengthen ethical engagement in clinical encounters (Natvik & Moltu, 2016). The aim of the current study was to describe lived experiences of adults who have been severely obese, have lost weight and sustained it over a period of at least 5 years.

Lifestyle change (diet and exercise modification), cognitive behavioural therapy, weight loss medications and bariatric surgery are treatment strategies for severe obesity (Bray, Frühbeck, Ryan, & Wilding, 2016). Weight loss surgery is the most effective intervention for long-term weight loss, but carries risk of mortality, complications and late effects, and health outcomes are largely unknown (Adams et al., 2012; Colquitt, Pickett, Loveman, & Frampton, 2014; Puzziferri et al., 2014). Surgery is not an option for all patients, because of individual preferences—for example, avoiding risks or advocating fat acceptance—or societal barriers, such as financial or health system barriers (NCD risk factor collaboration, 2016).

Modest weight loss, 5–10% of initial body weight, is sufficient to gain significant health benefits and prevent obesity-related illness (Goodpaster et al., 2010; Look AHEAD Research Group, 2010; Thomas, Bond, Phelan, Hill, & Wing, 2014; Wing & Hill, 2001). Whether modest weight loss is experienced as enough may depend on initial size, weight and health. Non-surgical weight loss initiatives for people suffering from severe obesity typically aim for a loss of 20% or more to achieve health benefits (Yumuk et al., 2015). Approximately 20% of those who obtain weight loss maintain it for one year or longer (Dombrowski, Knittle, Avenell, Araújo-Soares, & Sniehotta, 2014; Wing & Phelan, 2005). Many terminate weight loss efforts in an early phase, and weight regain is frequent (Christiansen, Bruun, Madsen, & Richelsen, 2007; Knowler et al., 2009; Look AHEAD Research Group, 2014; Ryan et al., 2010; Thomas et al., 2014). This means that long-term maintenance of weight loss via lifestyle changes is possible, but difficult (Frühbeck, Toplak, Woodward, Halford, & Yumuk, 2014; MacLean et al., 2015).

The National Weight Control Registry (NWCR) has identified successful weight loss maintainers and described their strategies, health behaviours and body weight trajectories since 1993. Participants with larger initial weight loss have been most successful in the long term (Thomas et al., 2014; Wing & Phelan, 2005). Successful participants regained more rapidly, but regained very little after five years, and heavier participants followed the same pattern (Thomas et al., 2014). Maintaining weight loss for 2 years or longer was associated with maintaining larger weight loss at 5 and 10 years. Initial behaviour changes correlated with outcomes 1 to 9 years later. Successful weight loss maintenance was associated with a range of factors and mostly with high levels of physical activity, low calorie and fat intake, high levels of restraint, low levels of disinhibition and self-monitoring (weighing) (Thomas et al., 2014). Stubbs et al. (2011) maintain that successful weight loss maintainers settle into a new profile of behaviours, attitudes and psychological profiles that essentially keep them in a state of vigilance.

Previous qualitative studies on non-surgical weight loss in overweight and obesity have provided valuable insights into weight loss and maintenance. Health-related behaviours, barriers and facilitators for weight loss, meanings of identity change and turning points in life have been described (Burke, Swigart, Turk, Derro, & Ewing, 2009; Byrne, Cooper, & Fairburn, 2003; Epiphaniou & Ogden, 2010; Garip & Yardley, 2011; Hindle & Carpenter, 2011; Lindvall, Larsson, Weinehall, & Emmelin, 2010; Ogden & Hills, 2008; Ogden, Stavrinaki, & Stubbs, 2009; Sarlio-Lähteenkorva, 2000; Stuckey et al., 2011). However, losing and maintaining weight from overweight/obesity and losing weight and maintaining weight loss from severe obesity might be significantly different phenomena, and qualitative studies of long-term weight loss maintenance after severe obesity are lacking. Stories of people who manage to lose weight and keep it off for the long term after severe obesity exist and are acknowledged, but are presented as exceptional, anecdotal success stories and are not analysed in detail (Freedhoff & Hall, 2016; Marzocchi, Cappellari, Dalle Grave, & Marchesini, 2011; Sharma, 2017). This means that as an experiential phenomenon, long-term weight loss after severe obesity is largely unexplored.

To gain new insight into sustained non-surgical weight loss after severe obesity, the people who have accomplished this (or not) and their experiential life is an important starting point. Research based in the first-person perspective can deepen current understandings of and meanings attached to long-term weight loss. Our research question was “What is it like for people with severe obesity to lose weight and keep it off for the long-term?”

## Methodology

To explore experiences of weight loss, we designed a qualitative study grounded in a phenomenological approach as described by van Manen (2014). Our ambition was to understand the ways in which weight loss following severe obesity incorporates the person’s living. Weight loss is a human phenomenon carrying lifeworld meanings. Lifeworld points to the world of experience, our particular world that we intimately know and take for granted (Husserl, 1954/1970). Ambiguity permeates our lifeworld as it is both all we know of and yet impossible to fully know (Merleau-Ponty, 1945/2012). In phenomenology, the lifeworld is both the source of and approach to possible experiences (van Manen, 2014). By taking a phenomenological approach, the current study aims to go beyond the taken-for-granted and give insight into essential meanings of long-term weight loss following severe obesity.

To get to a phenomenon’s meanings, the researcher must be attentive and open. Being open to the world as experienced and trying to “bracket” assumptions, lay understandings and scientific explanations are basic activities in our approach. These processes lead back to the phenomenon given in experience (van Manen, 2014). To see meanings and distinguish long-term weight loss after severe obesity from other phenomena, we engaged in reflection and draft writing. Getting access to lifeworld dimensions and existential meanings is a challenge, and doing justice to lived experience is a continuing concern throughout the research activities (van Manen, 2014).

The group of researchers behind this study comprises two physiotherapists, one nurse and one clinical psychologist, all with extensive experience in obesity research, both qualitative and quantitative. We have a particular interest in phenomenology, in existential and situated meanings of weight loss as a human phenomenon. The point of departure for our understanding as health care workers is that severe obesity can be harmful to an individual’s health and well-being. Severity of weight problems (size, co-morbidities) and sustainability of weight loss is important for health and in clinical encounters, and therefore provides an important context for studying successful weight loss. All researchers participated in planning the study, read transcripts, engaged in reflective dialogues about materials and contributed to writing this article. Three authors (E.N., C.M., M.R.) interviewed participants in depth and conducted analyses. The first author conducted five interviews and one follow-up interview, practised reflective writing and drafted this manuscript.

### Recruitment and participants

We sought experiences from persons who previously had a body weight categorized as severe obesity according to Body Mass Index (BMI) and currently had a body weight below severe obesity. People who had started losing weight at least 5 years prior to the study, and who had kept off at least 10% of their initial body weight for these years got a letter of invitation. People who had undergone weight loss surgery were not included. Previously, we have described long-term weight loss after bariatric surgery in several studies (Natvik, Gjengedal & Råheim, 2013, Natvik, Gjengedal, Moltu & Råheim, 2014, 2015). Results of long-term post-surgery experience studies, in particular that having bariatric surgery involves becoming a patient who needs medical care and follow-up, spurred interest in what long-term weight loss maintenance was like outside the context of surgery. We recruited participants with the assistance of experienced family physicians and weight loss experts in well-established commercial programmes. Ten people volunteered to participate. They were 8 women and 2 men aged between 27 and 59 with a median age of 37. The sampling strategy in this study is thus purposive (Malterud, 2001). The number and variation of participants was sufficient to produce a rich experiential material (van Manen, 2014). Table I presents the characteristic of the participant group.

**Table I.** Frequency table of participant characteristics.

Descriptor	Variable	Frequency
Gender	Male	2
	Female	8
		
Age	20–30 years	3
	30–40 years	2
	40–50 years	4
	50–60 years	1
		
		
Highest BMI	35–40	1
	40–45	3
	45–50	4
	50–60	1
	> 60	1
		
Current BMI	20–25	1
	25–30	5
	30–35	1
	35–40	3
		
Weight reduction %	20–30%	2
	30–40%	4
	40–50%	4
Relationship status	Married w. children	5
	Married	1
	Cohabitant w. children	1
	Cohabitant	1
	Single	2
Employment status	Full-time employed	6
	Self-employed	1
	Student	1
	Sick leave	1
	Retraining	1

### Ethical considerations

The Norwegian Committee of Ethics in Medicine approved the study, and the protocol was registered at the Social Science Data Service. Participants got written information about the study, its background, aims and specific information about interviews. We underscored voluntarily participation and guaranteed participants’ anonymity. Information was given by letter and telephone, and repeated in interviews.

### Conducting interviews

We conducted 10 in-depth interviews and 1 follow-up interview with a participant whom we asked to elaborate the first interview. All interviews were audio-recorded and transcribed verbatim for analyses. Interviews lasted on average 92 minutes, ranging from 59 to 124 minutes. We aimed to obtain experiences as lived through, rather than views and readymade opinions (van Manen, 2014). To create an atmosphere for attentive and open dialogues about what it is like to lose weight and maintain weight loss for the long term, participant and interviewer agreed on a suitable interview setting. We used a quiet space where interruptions could be avoided. Some preferred to be interviewed in a meeting room close to where they live or work, and others invited the interviewer into their homes.

Interviews began by breaking the ice, making space and taking place; for example, by finding a good way to sit, getting coffee and so on. The interviewer repeated the purpose and our interest in what it is like to engage in weight loss and weight loss maintenance for years after severe obesity. To make participants familiar with the interview situation and get contact, the researcher began each interview by collecting background information on age, education, employment and family situation, current weight, height and weight loss. Following this introduction, interviews centred on five topics: (a) The personal weight-journey; (b) Own body; (c) Habits and practices to maintain current weight; (d) Social life; and (e) Health and a good life. We introduced each topic with a broad question, such as “Can you tell about your weight changes throughout life until today?”

Aiming for concrete and detailed experiential descriptions, we prepared questions to get closer to particular events and situations, such as “Has your weight at any point been a threat your situation? Do you remember a particular event? Can you give an example?” Most participants talked about their weight loss processes in response to open questions. Before summing up the main dimensions in the conversation, the interviewer asked, “What do you think about the future?” Furthermore, participants were invited to add or deepen anything they felt like sharing about their weight journey, body weight and own body.

The participants grasped this opportunity to talk about weight loss experiences, and follow-up questions were not always needed. As there is a cultural interest in weight loss and strategies, healthy habits and practices, we concentrated on *experiential* accounts. We prepared to facilitate concrete and detailed descriptions and not to encourage stories told several times before. Preparations were useful in the interviews and were central to obtaining sufficiently rich experiential material. In-depth interviews provided possible experiences of weight loss for us to reflect on, interpret and write from (van Manen, 2014).

### Analysis

Analysis was inspired by phenomenological methodology as described by van Manen (1997, 2014). Researchers read transcripts, wrote reflective notes and engaged in reflective dialogues about each interview and the whole material. We aimed to grasp and describe a core meaning, an essential structure of sustained weight loss after severe obesity. Reflective dialogues included searching for and creating eidetic phrasings to capture and express the heart of the phenomenon. This way of approaching the textual material is termed holistic reading (van Manen, 2014). “Terminable and interminable”, reflecting a process ever vulnerable to being brought to halt, but that at the same time could never end, captured the eidetic meaning of the text as a whole, and an “essential phrasing” to continue our analysis from.

The first author continued the analysis by reading reflective notes already produced, rereading each interview and writing drafts. Interviews were reread with interest in particularly striking expressions and statements about losing weight for the long term and bearing the essential phrasing in mind. Moving back and forth between writing, rereading transcripts, reflective dialogues about the material and rewriting, themes started to emerge. The themes constitute the meaning structure of engaging in long-term weight loss after severe obesity, and consist of one essential and four related subthemes. “Never-ending story” expresses the most abstracted level of analysis of which the subthemes describe nuances and variations of losing weight and keeping it off. All themes are mutually related and interconnected. We chose to use participants’ experiential descriptions (quotes) in the subthemes, titled: “self-driven, but not alone—a transformed relationship to self and others”, “still different—joining new communities”, “distrusting one's own body—building systems and structures to lean on”, and “interrupted”.

## Findings

### A never-ending story

Lasting weight loss was the only way to get a second chance at life after severe obesity—a life the participants found worth living. A resilient decision to conquer one’s own weight issues was essential for establishing a profound lifestyle change. Deciding for lasting change meant so much more than making healthy choices and standing firm. Rather, it was a watershed moment in life. A deeply personal, perceptive and existential realization about one’s own situation and related to body weight. The large and heavy body no longer complied with a good life, nor had it done for a long time. Deciding to do something about it implied an ongoing engagement and commitment to changes—a sense of being fully immersed in profound change. Enduring changes. Incorporating changes. Adjusting changes. Participants put the decision to change at the very foreground of life itself, rather than it being one of many projects.

Losing and keeping weight off for the long term was a challenge requiring continuous attention and efforts. Losing weight implied rethinking meaningful aspects of life—relationships, one's own body, emotional life and personal values. Weight loss and weight loss maintenance were an epochal change. Sustained weight loss had no endpoint, yet was always easy to end. When following severe obesity, sustained weight loss was a never-ending story.

Five years into a stable, lower weight, a strong sense of not having got there still prevailed. Lifestyle changes were continuous, absorbing and touched the core of the participants’ being. After at least 5 years of living through severe weight issues, profound lifestyle changes and sustained weight loss, participants still kept forcing themselves to continue recreating and renewing their ways of living healthily. This constant labour inevitably affected personal identity.

Securing weight loss implied a heightened sensitivity towards oneself and others. Maintenance involved being alert, thinking and acting to overcome a multitude of challenges. Emotions, habits, urges and temptations could all threaten weight loss. Sensitivity following weight loss typically allowed opening up, establishing or sustaining relationships to oneself and others. For the formerly large people, the life they had longed for became possible and real. This was a paradox. Committing to change was very much a lone commitment to oneself, yet being successful inevitably involved others. Other people might both help and threaten the process. Family and friends, workplaces and health care professionals being able to adjust or refrain from jeopardizing the weight loss process played a crucial role in long-term weight loss and change. Long-term weight loss presupposed a commitment to making changes. To endure, refine and make new changes, and always by keeping the starting point in mind. To leave severe obesity forever, the formerly large body and life could not fully be left.

### Self-driven, but not alone—a transformed relationship to self and others

Deciding to lose weight for the long term was a meaningful and deeply personal turning point. The decision was pivotal for staying in and recommitting to pervasive and encompassing change every day for 5 years or more. Weight loss processes were highly self-driven. Most participants only told others about the decision when they felt more secure that they would carry on with it, when the decision had turned into successful action. One woman, who was a former athlete, said:

One day, I thought [stops]. I was going to watch a hockey game in a city nearby. We parked at the bottom of the hill, you know, down by the [stops]. It’s a quite steep uphill to the ice arena. I couldn’t keep up with the others up that hill, and [thought], “I cannot live like this, I must do something. I’m going to work as a health care professional, which is quite a physical job.” … They [at home] did not really believe in me. I went for a walk with my mother. I guess I had been on the weight loss programme for a year. I had started noticing my clothes becoming too big, so I said, “Now, I must start getting rid of my clothes. I’ll go home and clear my closet.” Then she said to me, probably without thinking, “Well now, there is no point in getting rid of any clothes, soon you will need them again. You’ll put the weight back on when you have finished the course.” …. I really had to show them.

Profound life changes aiming at long-term weight loss maintenance was a complex journey. Always being self-aware, self-observing and ready to adjust. Although the large body was history, it lingered in awareness. Accepting the large body that one used to be was significant in embodying weight loss, making changes and continuing them. Losing weight and maintaining it over time entailed a new relationship to different aspects of oneself simultaneously. The new self, the old self, and the image that presented itself in the mirror. One man, who had lost 100 kilos, said:

It lasts for a long time, that I have been [short pause] very much larger. I guess I don’t have the complete picture [short pause]. My body image is not fully updated [short pause]. My body weight and -image have not arrived at the same time, to put it that way. It lingers a little. Not that I stand in front of the camera that often, but what more can I say? [Short pause]. After all that exercise, I feel fit [short pause]. … When I have lost weight, I can see it. I mean, it might not be there, but I see it. At least I feel that I can see it in the mirror. If I have had a hard time, I think I can see that in the mirror too. It is as if I can see all the faults in difficult times. When things are going well, I see all good things … but mostly the negative in times of difficulty. … It is as if I have no worries when things go [well]. It’s like being on a wave, and that’s what I was, at least for three years. Just a flow of uplifting things kept happening to me.

Weight loss involved reflecting on past and present situation, practising new ways of living and incorporating a wide variety of emotions. Self-awareness now implied a growing and personal sense of emotions. Participants were ambivalent to emotional aspects of weight loss and were careful of going too deeply into problematic experiences. Difficult emotions might disturb a process they needed to continue. Participants balanced the amount of painful emotions allowed into the process with the need to do what long-term weight loss demanded:

I think I missed a lot during the process. I didn’t take time to notice what I felt. It was just a joyride …. Back then, I was “Bea” in the middle of a huge and successful super project and everything was awesome. I didn’t need anything else. I was on top of the world …. Being me now is this process of trying to build a new identity. That’s what I need to do. I’m no longer that big, fat person. This has to do with seeing myself [stops]. However, I’m always reminded [about the past]. Yet I don’t want to forget about it either, right? It is part of me.

Having experienced what being (too) large means seemed to add existential quality to weight loss. Insights connected to previous life intertwined with the present. A space for feelings cleared. Sadness, anger, self-compassion, warmth and generosity towards oneself and the large body gone were allowed their presence:

I was very large, and I didn’t have any thoughts about starting a family, or finding a boyfriend. Which really was [what I wanted]. In the end, I just dropped it. Being so large, I couldn’t think about that at all. In a way, I lost [stops]. Having children was nothing to think about at that time, but when things [lifestyle change] started to work out, I got hope. … I often think about it, and almost have to pinch my arm, “Is this me?” So much has happened in the years after I finished [the weight loss programme]. … It didn’t take long before I started trying to find a boyfriend and moving on. It feels as a long time ago, even if it has not gone that many years [clearing throat]. So many good things have happened afterwards. From [clearing throat] feeling so low back then, to how I feel now. That’s my motivation for not putting weight back on.

Weight loss had opened the participants’ lives up for other people, which was a gain. Others were crucial in maintaining the process. Although changes were highly and primarily self-driven, continuing support was essential. Participants needed to be surrounded by others who were willing to adjust to create and live a totally changed life. They had experienced family, friends and colleagues changing or continuing as before, acting rather indifferently or positive and patiently:

My husband is slim and can eat all he likes, so it was a bit difficult for him to start this [process] and understand it. I started, we made a plan for how to do it, and that made it easier for him. He started to understand and saw the results. He says that I’m a different person now. More present, more awake and wanting to do things. Yet it’s difficult for me to describe how hard it really is.

Employers who understood and supported the weight loss processes meant a lot. They made it possible for participants to combine working-life and “weight-life”. Participants deeply appreciated health care professionals cheering when achieving goals and successes. People who kept on encouraging them while encountering hindrances or suffering defeats. Not all people understood what living with major weight loss meant and involved for the one leaving severe obesity. There was an impression that some might find major changes leading away from weight problems hard to comprehend and tolerate:

Many did not understand the change at all … . I had to explain myself, and of course, when I turned down invitations five times in a row, I didn’t get the sixth one. It’s a bit like that [laughs]. They have accepted it, and I hope they can see why I have made these choices. … They [friends] are not large, just a little round and have tried exercise, but didn’t make it. I feel that they were never happy for my success; no one ever said anything like “You made it.” … I didn’t get much understanding, and I needed to talk to my supporting team [local nurses, GP, family]. They gave me the understanding I needed.

Weight loss and lifestyle change could reconnect the previously large person and the social world, in that they increasingly trusted and reached out to each other. But lifestyle changes could be all-consuming. Concentrating on weight loss could come between the person and other people, and not committing to relationships resulted:

I don’t go out, I work a lot and I exercise. At one point, I would like to find someone to share my life with, but I have no hurry. I’m fine the way things are right now. I live the life I want to live. My focus is on lots of exercise, competitions and races. But suddenly it might happen. This has to do with everything going on right now. But on New Year’s Eve, I will probably think “Why don’t I have someone to celebrate with?”

To understand oneself in new and meaningful ways was clarifying. To lose weight and keep it off through self-driven efforts and change evoked a sense of personal strength and self-confidence. Embracing new ways of living, feeling healthier and happier became something worth sharing. Five years into successful weight loss processes, relationships were a topic for all participants. Engaging in a close relationship, or longing for someone to love. Some relationships had grown deeper, and some were lost.

### Still different—joining new communities

Life after severe obesity had to be lived quite differently compared to the lives of people who were not leaving severe obesity. Losing weight meant coming closer to “the ordinary” with regard to body size, but remaining different in important ways. Strong commitment to diet and exercise on a daily basis for years is a certain way of life. To avoid regain, participants continued thoughtful planning and were much less flexible to enjoy life in unreflected and spontaneous ways, which is remarkably different from how most people live. Navigating the modern western culture of plenty of food and easy access with stamina, control and moderation was an extremely hard task. Succeeding to lose weight and live healthily and controlled in the long run was personally and socially prestigious:

It’s difficult with food. I’ve had to look into what my problem with eating and weight is, and that’s sweets. I never ate too much. I eat a normal dinner, or maybe less but I can start snacking right afterwards. I can eat at least the same amount of calories when snacking. I can have it, even when I feel full. For me, the problem is the cravings for sweets and the energy boost that I feel in my head. … So [sigh]. All the time I’ve been like “Why? Why is it like this? Why, why can’t I eat normally”? Underlying emotional stuff started the whole thing [severe obesity]. But I found a way to tackle it physically, by eating differently. Then I can’t eat stuff making my blood sugar fluctuate. I can’t eat that anymore, because I get that physical reaction. I need to eat a special and different diet to keep my blood sugar stable. I can do that, even if it is a lot of [short pause]. It’s a little tricky. It’s fine when I’m at home, but the world is not set up for my way of living.

To restore normality with regard to body size, participants had committed to a life of difference. Different habits, food routines and exercise gave an outsider position to social life. Being different like this appeared as admirable or enviable to others. Not worrisome, condemnable or disgusting as they had experienced that the large body had been. That is, weight loss and change gave new and meaningful opportunities to engage with the social world. Yet valuable encounters with others were at stake in the continuous worry about regain. Weight regain meant losing exactly this newly found and deeply important social potential. Living differently remained very challenging.

Finding others or communities who valued and shared similar lifestyle commitments became important. Most participants had built a range of new relationships with people who shared their interests. They joined others who were, for example, developing cooking skills, carrying out a low carbohydrate diet or practising vigorous exercise for competitions:

I lost my friends when I went into this bubble [lifestyle change] and became rather asocial. I could no longer join in. Or maybe I could, but in my situation, I couldn’t take part in girls’ nights or enjoying myself like that. I knew I needed to sleep a lot to be ready for exercise next day. I needed to focus on that. I realize that I almost only have older friends in my life after I made the changes. Friends aged 35 and up, because they are in the age of crisis and need to start exercising [laughing]. I hang out with them now [laughs]. I need to be with people who are in the same phase or who are more like me now.

By losing weight, enduring lifestyle change and maintaining weight loss, participants stood out from the crowd for other reasons than conquering severe obesity and becoming slimmer. In fact, they managed things so many have tried and failed to do. Life after severe obesity demanded being constantly alert, staying on track and protecting oneself from the surroundings. The culture of always-present food, easy access to plenty of snacks and great opportunities to stay inactive demanded steady navigation. The cultural push towards weight gain has made it a phenomenon to which many adults can relate. The participants and their achievements were rather unique and attracted others’ attention and admiration. Such surroundings played a part in evoking memories of what life used to be like:

I’m into running, cycling, triathlon and big races. I’m on that wave, and I need to lose a few kilos to become better at that. … I still work on how others outside my home [laughs] see me. I have some issues. I have stretch marks and still a flabby stomach. I have something to work on, but it is not like before. Back then, I waited until everybody else had finished in the public shower before I finally could enter. I use public showers now, but I am not standing in the middle of the room towelling myself dry.

According to participants, body weight rarely came up in conversations while they were large. Others used to meet their excess weight and problems attached with silence. After weight loss, body weight reappeared as an inevitable and very interesting topic. Problematic weight—for example, weight cycling—remained silent. Weight gain has become a topic of debate among lay people, public health advocates and health care providers, in politics and in the wider popular culture. In this light, the participants accomplishing weight loss and a healthy lifestyle for life expressed one of the glories of the modern western world. Through long-term weight loss after severe obesity, they expressed a unique mastery of a special body of knowledge:

The [lifestyle] changes are only positive for me, and I am very engaged in it. Change has done me so [much] good, and so I would like to engage other people. I try to spread a little healthiness [laughs]. I try to motivate people to get up from the couch. Many have asked me for advice on food and eating, because they can see what I have done. I like to talk about it.

Participants had started *seeing* others struggling with severe obesity. Seeing people striving against severe obesity touched them, and echoed their own processes. They wanted to help, and expressed a sincere enthusiasm to share, include and support others in making important life changes. “Lived through” insights sparked initiative and interest beyond one’s own process. Most participants wished to work professionally with lifestyle change, and some already did.

Switching from severe obesity to a healthy and lighter life was all encompassing and constantly ongoing. In social life, participants experienced that profound change seemed to act as reappearance and restoration of human dignity and moral life. Concentrating on weight, diet and exercise for the long term was imperative to escape severe obesity, but risks followed. New structures could turn into obsession, illness or total exhaustion. To stay at a normal weight, everyday life had to be structured and lived differently. Different from the previous life and from the lives of many others, yet balancing not too far away from the norm.

### Distrusting one’s own body—building systems and structures to lean on

Experiences of being large motivated and forced change, and led to a healthy and carefully planned structure in everyday life. Monitoring and weight control had become unavoidable. Establishing objective checkpoints in everyday life and sticking to them over time was important for building a structure to lean on, such as weighing oneself, measuring food portions sizes, carefully considering daily amounts of carbohydrates, proteins and fat and calculating energy expenditure. Participants checked up on themselves and created predictability in their lives in different ways, but their strategies always involved a personal structure for food and exercise, even 5 years or more into stable lower weight:

I get up early in the morning, and have oatmeal porridge for breakfast. I often use the kitchen scales and portion out, to check the amount. We are lucky to have a canteen at work, and I eat salad. Every Friday, I have some cheese and pasta in my salad. It’s a celebration, and I look forward to it. We have ordinary dinners, typically spaghetti, chicken or fish, and we weigh it. I sleep for half an hour before I exercise. Spinning, jogging, swimming or strength training; I exercise every day and extra on the weekends; 2-hour workouts or hiking in the mountains on Saturdays and Sundays. We always have a meal in the evening.

Self-monitoring had become part of the participants’ everyday lives, and created both confidence and a certain pressure. Participants varied in what actions they emphasized to keep and to keep on with changes. Some concentrated fully on specific goals ahead, typically oriented towards physical activity and competitions:

In the beginning of the process, I kept thinking, “Why haven’t I always done this?” I’m competitive and very stubborn, and I like to do it this way: “From now, I will do this for 100 days in a row.” So [breathing out], I was driven by an intense chasing of not having calendar gaps … and I was largely going on the autopilot. I felt very restless coming home from work, with many hours left before bedtime. I had [strong emphasis] to do something.

The strategy of making one’s body able to move and perform, and in that sense become its own asset for weight control, was essential. Exploring and taking advantage of one’s own bodily potential and capability connected to joy, strength and invincibility. Experiencing their own bodies as capable and fit gave the participants access to new social spaces and physical activities—and courage to enter and take part. This contrasted social, emotional and practical hindrances attached to the large body. Performances and achievements related to physical activity strengthened participants’ confidence in themselves as bodily beings. Nevertheless, experience with severe obesity and failed attempts to lose weight meant that the body could not be trusted. The body needed to be controlled. In this sense, lifestyle change and weight control became bodily performance and vice versa. Meanings attached to one’s own body changed dramatically with weight loss, performance and conquering new fields:

I exercise a lot and participate in Triathlons and so on. I need to lose a few more kilos to improve. I actually beat my physician in a cycling race [laughs]. … It’s a little funny, because I was always the biggest, but at the same time the best girl in many sports during childhood. I was always very active, never at home … . I knew what I could do. I knew the difference between a stroll in the park and pacing yourself so hard uphill, lying on the ground and barely able to talk to anyone. Not everyone knows how to exhaust yourself. I knew what was good enough and what wasn’t. I’m not afraid of being breathless. You can always push a little harder when exercising. A workout is not 15 minutes, but 1 hour, 1 hour and a half. I remember how soon I felt fit again, not comparing myself with others, but for myself, I was fit. My goal is to run a marathon and weigh 72 kilogrammes.

Targeting goals, moving forward, pushing the body and exceeding personal limits was rewarding and satisfying, but gave little space for reflection. Participants mobilized different personal resources. Sadness, exhaustion, loneliness and despair were part of the process, but not necessarily dwelled on. Participants seemed to suppress feelings related to resignation, but their own body’s fragility was inevitably present. Identifying with the large body (gone) seemed to bridge past and present self-understanding.

### Interrupted

Five years of lifestyle change involved holidays, illness, injuries, childbirth and other life events. Life events forced pauses or required adjustments in well-controlled eating practices and exercise. For persons who had overcome severe obesity, holidays and other extraordinary periods occasionally interrupting their new, healthy and preferred ways of living were troublesome. On this matter, participants expressed ambivalence. Temporarily cutting some slack regarding weight and ways of life was unavoidable in the long term. Cutting slack inescapably led to rapid weight gain. Setbacks demanded strong efforts and caused profound worry. Participants needed to work hard to get back into routine and structure. Losing regained weight was a struggle. On the other side, temporary attempts to “let go” seemed to play an important role in negotiating degrees of freedom. Testing limits, particularly with regard to eating, was part of long-term weight loss maintenance after severe obesity. Yet, participants needed to conquer severe obesity forever. Gaining weight might put the whole process at risk. Pleasures of freedom were not worth weight regain, according to participants’ experience:

I admit that I panicked [with emphasis] when stepping on the scales after Christmas. I had gained so much in such a short while; I was frantic [with emphasis]. I did not want to do it anymore, right. It was just fine going back to normal eating. Not eating carbohydrates has become normal for me now, although I sometimes eat some of it. … I had to sort out what was me. What did I do for others, how did I function? Everything that I did is what I planned to do. I have done my best, and when I have failed, it’s ok. I’m a human being. However, that’s no excuse to let everything go and just let it slide … . I’m allowed to be a human being. If I eat a slice of cake, everything is not messed up. I just have to get back on track right away.

Continuing the process of weight loss was personally and existentially valuable. Participants dreaded any prospect of it ending. Pleasant experiences, such as indulging in a full traditional celebration of Christmas, eventually end. Participants expressed that returning to previous pleasures might not be that enjoyable or important. Interruptions like a traditional celebration seemed to clarify the personal life changing process and meanings attached. Having faith in the process and living slightly relaxed was possible and to some extent necessary. Participants were always accompanied by high risk of relapse and regain. The thought of returning to severe obesity was intolerable, yet it was extremely easy to slip:

I fractured my leg a year ago, and have had low back pain and a painful knee. I could barely walk for four months, and needed crutches to move. I became very inactive and gained a few kilos. I caught myself thinking that my weight might rise, although I have decided that it’s never [with emphasis] going to happen. It was a bad time. I was terribly bored and it was a hard testing on my patience. I just missed walking [with emphasis], moving my body, and to do whatever I wanted to. … I tried to continue, went to the gym every day, but it was a struggle. I have a new lifestyle, which I really enjoy, and [when injured] it was taken away from me.

Leaving severe obesity demanded effort and thoughtfulness through and through. Following severe obesity, long-term weight loss was expressed as a success. Participants incorporated being successful through profound change. Sustained weight loss after severe obesity meant a never-ending story. In that sense, losing weight for the long term implied a turning point, making and living with profound changes, and never stopping to do so:

It is a lifelong project, and I think about it all the time. There is no fear of going back where I was, because I am so determined that it’s not going to happen. Nevertheless, I think about it all the time … . It feels like a job in which I cannot have a break.

## Discussion

Losing weight and maintaining weight loss after severe obesity had become the foreground in life for the people who had decided to go through with it. Self-driven changes and actions provided and secured weight loss, and were intensely meaningful and challenging for the participants to continue. The process incorporated their intentions, projects and interconnection with other people and the surrounding world. Carrying out and maintaining weight loss after severe obesity was a profound, bodily and existential experience.

Thoughts, emotions and material qualities of the body are inseparable, and always connect with the social world (Merleau-Ponty, 1945/2012). Feelings, thoughts, actions and encounters with others begin with the body, express the body, and carry meanings. Bodily and subjective identity are inseparable, and *lived body* expresses this ambiguity or double status of the body. The lived body is both experiential and our expression to the world, showing personal style, mood, emotions and intentions, as well as more objective bodily states, such as gender, ethnicity and body size. In the everyday experience of a healthy body, the subjective and objective aspects are in agreement, and the lived body remains background (Carel, 2008).

In our findings, the changing body and continued weight loss seem incompatible with the spontaneous and taken-for-granted body, and the objectified body is insufficient to contain the full process of change. Keeping weight off and practising healthy eating and exercise was crucial, but never enough. A structural change of the lived body, in how the participants experienced, responded and acted in everyday life, was central to the phenomenon. This means that participants incorporated bodily changes and the ongoing changes to maintain weight, albeit not by an instrumentalist approach: their journeys seemed like the people they had become. In this incorporated being, recognition and awareness of the material reality of one’s own body was paramount. Importantly, this materiality was inseparable from other aspects of one’s being, such as values and identity, relationships and sociality, longings and suffering. The objective and subjective body coexisted in the weight loss maintenance processes after severe obesity. In this respect, weight loss maintenance is importantly different from an illness experience. Illness experience involves a split between the subjective and objective body, and is often connected to alienation (Carel, 2008; Svenaeus, 2000; Toombs, 1993).

Losing weight and keeping it off after severe obesity seem to have more in common with the philosophy of the recovery tradition in the field of mental health. Personal recovery, in their usage, is defined as “a deeply personal, unique process of changing one’s attitudes, values, feelings, goals, skills and/or role“ (Anthony, 1993, p. 15), in finding meaningful ways to live with challenges rather than aiming to get rid of them. However, defining personally meaningful ways to live with challenges presupposes a certain degree of autonomy.

Greaves, Poltawski, Garside, and Briscoe (2017) synthesized the results from 26 qualitative studies of weight loss maintenance, including participants who had undergone weight loss surgery, who worked on non-surgical weight loss strategies, who were both successful and unsuccessful at maintaining weight loss. Weight loss magnitude and stability over time varied across the 710 participants, suggesting they analysed a cluster of phenomena out of which the phenomenon we have studied might be one. Building their resulting model around constant cognitive and behavioural control of tension, Greaves and colleagues suggested some differences between people who regain and people who maintain with regard to identity and temporality. They reported that weight loss maintenance constituted a “constant battle” (Greaves et al., 2017, p. 151). Weight loss maintenance entailed going beyond resistance and tensions and taking action to reduce or eliminate them, whereas weight regain seemed connected to handling weight loss behaviours as temporary measures (p. 155). Discussing this difference, the authors indicated a process of evolution into a new person for those who maintain weight, with a different outlook, social life, priorities, leisure pursuits and career (p. 156). Our study allows for a thorough analysis of this particular phenomenon, echoing Greaves and colleagues’ results and elaborating on the process of change beyond habits, behaviours and lifestyles (Greaves et al., 2017).

Continuing the line of thought stemming from autonomy and personal recovery, our findings echo results from a qualitative study investigating women’s experiences of falling ill with fibromyalgia, being in recovery and staying healthy (Grape, Solbrække, Kirkevold, & Mengshoel, 2015). After fibromyalgia, being healthy demanded major and constant efforts to maintain the body and profound changes in everyday life. Ongoing bodily awareness, analysing bodily signals, eating healthily and balancing exercise and relaxation was necessary to avoid illness. According to Grape and colleagues’ findings, following fibromyalgia, life will never be the same, even after recovery (Grape et al., 2015).

In traditions such as the field of mental health and fibromyalgia, where shadows of health challenges often demand lifelong adjustment, recovery traditions building on meaning creation, identity and values have been developed over years. However, the dominant scientific perspective on obesity seems not to capture the *existential*, *autonomous* and *personal* experience of losing weight and maintaining weight loss emergent in our findings. Interventions and measures of effects as solutions to the problem of obesity dominate obesity research. Various forms of restricted diets, increased levels of physical activity and strategies from cognitive behavioural therapy are frequent research topics, with emphasis on weight loss, energy consumption, activity levels, biomedical parameters/blood and quality of life. The inherent epistemology of such interventions is dominantly instrumentalist, meaning that the end-point outcome for a part of the person attains the centre stage. Yet life is not an outcome, it is a process. Referring back to the differences between maintainers and regainers reported in Greaves et al. (2017), regainers had more of a tension-release structure with micro-endpoints attained and lost, whereas maintainers saw the needed changes as their new being as people. Changes that would never end because they would become them.

Indeed, when moving from knowing what was good for them to doing it, the participants in the current study drew on common weight loss strategies, but importantly *not* as if they took part in a lifestyle programme that health professionals or lifestyle experts had planned for them and whose goals they adapted. The participants were not primarily subject to an intervention or institution. Rather, they incorporated weight loss as they had incorporated their excess weight. Painful obesity-related issues and longing for a better life had pushed participants towards dramatic life changes and altering their own bodies. Initiating and living through weight loss made them explicitly aware of themselves as bodily subjects. The current study points to the existential domain, and that processes needed to change lifestyle and lose weight for the long term are deeply personal. The commitment to living a changed life was a decision of the self, and an autonomous commitment to one’s own life.

## Methodological considerations

Starting from the lifeworld, we have presented a context-sensitive and meaning-oriented description of sustained weight loss after severe obesity. In phenomenological research, validity is associated with the originality of insights and the quality of the analytic processes shown in the study (van Manen, 2014). Furthermore, validity relates to the questions asked, the support of participants in returning to their experiences, understanding the difference between content, meanings and the meaning structure of a phenomenon (van Wijngaarden, van Der Meide, & Dahlberg, 2017). We hold that our findings are strong, and the analysis of underlying meaning structures of weight loss is transparent to readers, including participants’ nuanced descriptions. However, meanings are ambiguous and tentative, leaving this text open for appraisal, questioning and expansion.

Note 1: Severe obesity is defined as having a Body Mass Index ≥40 or having a BMI ≥ 35 and obesity-related illness (World Health Organization, 2017b).

## References

[CIT0001] Adams T. D., Davidson L. E., Litwin S. E., Kolotkin R. L., LaMonte M. J., Pendleton R. C., … Hunt S. C. (2012). Health benefits of gastric bypass surgery after 6 years. *JAMA: Journal of the American Medical Organization*, 308(11), 1122–12. 10.1001/2012.jama.11164 PMC3744888 22990271

[CIT0002] Anthony W. A. (1993). Recovery from mental illness: The guiding vision of the mental health service system in the 1990s. *Psychosocial Rehabilitation Journal*, 16, 11–23.

[CIT0003] Bray G. A., Frühbeck G., Ryan D. H., & Wilding J. P. H. (2016). Management of obesity. *The Lancet*, 387(10031), 1947–1956. 10.1016/S0140-6736(16)00271-3 26868660

[CIT0004] Brewis A. A. (2014). Stigma and the perpetuation of obesity. *Social Science and Medicine*, 118, 152–158. 25124079 10.1016/j.socscimed.2014.08.003

[CIT0005] Burke L. E., Swigart V., Turk M. W., Derro N., & Ewing L. J. (2009). Experiences of self-monitoring: Successes and struggles during treatment for weight loss. *Qualitative Health Research*, 19(6), 815–828. 19365099 10.1177/1049732309335395 PMC2855299

[CIT0006] Byrne S., Cooper Z., & Fairburn C. (2003). Weight maintenance and relapse in obesity: A qualitative study. *International Journal of Obesity and Related Metabolic Disorders*, 27(8), 955–962. 12861237 10.1038/sj.ijo.0802305

[CIT0007] Carel H. (2008). *Illness: The cry of the flesh*. Durham, England: Acumen Publishing.

[CIT0008] Christiansen T., Bruun J. M., Madsen E. L., & Richelsen B. (2007). Weight loss maintenance in severely obese adults after an intensive lifestyle intervention: 2- to 4-year follow-up. *Obesity*, 15(2), 413–420. 17299115 10.1038/oby.2007.530

[CIT0009] Colquitt J. L., Pickett K., Loveman E., & Frampton G. K. (2014). Surgery for weight loss in adults. *The Cochrane Database of Systematic Reviews*, 8. doi:10.1002/14651858.CD003641.pub4 PMC9028049 25105982

[CIT0010] Dombrowski S. U., Knittle K., Avenell A., Araújo-Soares V., & Sniehotta F. F. (2014). Long term maintenance of weight loss with non-surgical interventions in obese adults: Systematic review and meta-analyses of randomised controlled trials. *BMJ: British Medical Journal*, 348. doi:10.1136/bmj.g2646 PMC4020585 25134100

[CIT0011] Epiphaniou E., & Ogden J. (2010). Successful weight loss maintenance and a shift in identity: From restriction to a new liberated self. *Journal of Health Psychology*, 15(6), 887–896. 20472607 10.1177/1359105309358115

[CIT0012] Freedhoff Y., & Hall K. D. (2016). Weight loss diet studies: We need help not hype. *The Lancet*, 388(10047), 849–851. 10.1016/S0140-6736(16)31338-1 27597452

[CIT0013] Frühbeck G., Toplak H., Woodward E., Halford J. C., & Yumuk V. (2014). Need for a paradigm shift in adult overweight and obesity management - an EASO position statement on a pressing public health, clinical and scientific challenge in Europe. *Obesity Facts*, 7(6), 408–416. 25503968 10.1159/000370038 PMC5644793

[CIT0014] Garip G., & Yardley L. (2011). A synthesis of qualitative research on overweight and obese people’s views and experiences of weight management. *Clinical Obesity*, 1(2–3), 110–126. 25585576 10.1111/j.1758-8111.2011.00021.x

[CIT0015] Goodpaster B. H., DeLany J. P., Otto A. D., Kuller L., Vockley J., South-Paul J. E., … Jakicic J. M. (2010). Effects of diet and physical activity interventions on weight loss and cardiometabolic risk factors in severely obese adults. A randomized trial. *Jama-Journal of the American Medical Association*, 304(16), 1795–1802. 10.1001/jama.2010.1505 PMC3082279 20935337

[CIT0016] Grape H. E., Solbrække K. N., Kirkevold M., & Mengshoel A. M. (2015). Staying healthy from fibromyalgia is ongoing hard work. *Qualitative Health Research*, 25(5), 679–688. 25387910 10.1177/1049732314557333

[CIT0017] Greaves C., Poltawski L., Garside R., & Briscoe S. (2017). Understanding the challenge of weight loss maintenance: A systematic review and synthesis of qualitative research on weight loss maintenance. *Health Psychology Review*, 11(2), 145–163. 28281891 10.1080/17437199.2017.1299583

[CIT0018] Gudzune K. A., Bennett W. L., Cooper L. A., & Bleich S. N. (2014). Patients who feel judged about their weight have lower trust in their primary care providers. *Patient Education and Counseling*, 97(1), 128–131. 25049164 10.1016/j.pec.2014.06.019 PMC4162829

[CIT0019] Hindle L., & Carpenter C. (2011). An exploration of the experiences and perceptions of people who have maintained weight loss. *Journal of Human Nutrition and Dietetics*, 24(4), 342–350. 21414044 10.1111/j.1365-277X.2011.01156.x

[CIT0020] Husserl E. (1954/1970). *The crisis of European sciences and trancendental phenomenology. An introduction to phenomenological philosophy*. Evanston, IL: Northwestern University Press.

[CIT0021] Knowler W. C., Fowler S. E., Hamman R. F., Christophi C. A., Hoffman H. J., Brenneman A. T., … Nathan D. M. (2009). 10-year follow-up of diabetes incidence and weight loss in the diabetes prevention program outcomes study. *Lancet*, 374(9702), 1677–1686. 19878986 10.1016/S0140-6736(09)61457-4 PMC3135022

[CIT0022] Lewis S., Thomas S. L., Blood R. W., Hyde J., Castle D. J., & Komesaroff P. A. (2010). Do health beliefs and behaviors differ according to severity of obesity? A qualitative study of Australian adults. *International Journal of Environmental Research and Public Health*, 7(2), 443–459. 20616984 10.3390/ijerph7020443 PMC2872289

[CIT0023] Lindvall K., Larsson C., Weinehall L., & Emmelin M. (2010). Weight maintenance as a tight rope walk - a grounded theory study. *BMC Public Health*, 10. doi:10.1186/1471-2458-10-51 PMC2835685 20122140

[CIT0024] Look AHEAD Research Group (2010). Long-term effects of a lifestyle intervention on weight and cardiovascular risk factors in individuals with type 2 diabetes. *Annals of Internal Medicine*, 170(17), 1566–1575. 10.1001/archinternmed.2010.334 PMC3084497 20876408

[CIT0025] Look AHEAD Research Group (2014). Eight-year weight losses with an intensive lifestyle intervention: The look AHEAD study. *Obesity*, 22(1), 5–10. 24307184 10.1002/oby.20662 PMC3904491

[CIT0026] MacLean P. S., Wing R. R., Davidson T., Epstein L., Goodpaster B., Hall K. D., … Ryan D. (2015). NIH working group report: Innovative research to improve maintenance of weight loss. *Obesity*, 23(1), 7–15. 25469998 10.1002/oby.20967 PMC5841916

[CIT0027] Malterud K. (2001). Qualitative research: Standards, challenges, and guidelines. *The Lancet*, 358(9280), 483–488. 10.1016/S0140-6736(01)05627-6 11513933

[CIT0028] Malterud K., & Ulriksen K. (2011). Obesity, stigma, and responsibility in health care: A synthesis of qualitative studies. *International Journal of Qualitative Studies on Health and Well-Being*, 6(4). doi:10.3402/qhw.v6i4.8404 PMC3223414 22121389

[CIT0029] Marzocchi R., Cappellari D., Dalle Grave R., & Marchesini G. (2011). Massive weight loss without surgery in a super obese patient. [journal article]. *Obesity Surgery*, 21(4), 540–545. 19841994 10.1007/s11695-009-0011-8

[CIT0030] Merleau-Ponty M. (1945/2012). *Phenomenology of perception* (2nd ed.). New York, NY: Routledge.

[CIT0031] National Institute for Health and Care Excellence (2006). *Obesity guidance on the prevention, identification, assessment and management of overweight and obesity in adults and children*. London, England: NICE Retrieved from http://guidance.nice.org.uk/CG43/Guidance/Section/5b/pdf/English 22497033

[CIT0032] National Institutes of Health (2012). What are the health risks of overweight and obesity? Retrieved 05.5.2015, from http://www.nhlbi.nih.gov/health/health-topics/topics/obe/risks

[CIT0033] Natvik E., Gjengedal, E., & Råheim, M. (2013). Totally changed, yet still the same: Patients’ lived experiences 5 years beyond bariatric surgery. *Qualitative Health Research*, 23(9), 1202–1214. 23921810 10.1177/1049732313501888

[CIT0034] Natvik E., Gjengedal, E., Moltu, C., et al. (2014). Re-embodying eating: Patients’ experiences 5 years after bariatric surgery. *Qual Health Res*, 24(12), 1700–1710. 25156217 10.1177/1049732314548687 PMC4232339

[CIT0035] Natvik E., Gjengedal, E., Moltu, C., et al (2015). Translating weight loss into agency: Men’s experiences 5 years after bariatric surgery. *International Journal of Qualitative Studies on Health and Well-Being*, 10, 27729. 26066518 10.3402/qhw.v10.27729 PMC4462825

[CIT0036] Natvik E., & Moltu C. (2016). Just experiences? Ethical contributions of phenomenologically-oriented research. *Scandinavian Psychologist*, 3, e17.

[CIT0037] NCD risk factor collaboration (2016). Trends in adult body-mass index in 200 countries from 1975 to 2014: A pooled analysis of 1698 population-based measurement studies with 19·2 million participants. *The Lancet*, 387(10026), 1377–1396. 10.1016/S0140-6736(16)30054-X PMC7615134 27115820

[CIT0038] Ogden J., & Hills L. (2008). Understanding sustained behavior change: The role of life crises and the process of reinvention. *Health*, 12(4), 419–437. 18818273 10.1177/1363459308094417

[CIT0039] Ogden J., Stavrinaki M., & Stubbs J. (2009). Understanding the role of life events in weight loss and weight gain. *Psychology Health & Medicine*, 14(2), 239–249. 10.1080/13548500802512302 19235083

[CIT0040] Prospective Studies Collaboration (2009). Body-mass index and cause-specific mortality in 900 000 adults: Collaborative analyses of 57 prospective studies. *The Lancet*, 373(9669), 1083–1096. 10.1016/S0140-6736(09)60318-4 PMC2662372 19299006

[CIT0041] Puhl R. M. (2011). Bias, stigma and discrimination In Cawley J. (Ed.), *The Oxford handbook of the social science of obesity* (pp. 553–571). New York, US: Oxford Universty Press.

[CIT0042] Puhl R. M., & Heuer C. A. (2009). The stigma of obesity: A review and update. *Obesity*, 17(5), 941–964. 19165161 10.1038/oby.2008.636

[CIT0043] Puzziferri N., Roshek T. B. Iii, Mayo H. G., Gallagher R., Belle S. H., & Livingston E. H. (2014). Long-term follow-up after bariatric surgery: A systematic review. *JAMA: Journal of the American Medical Organization*, 312(9), 934–942. 10.1001/jama.2014.10706 PMC4409000 25182102

[CIT0044] Ryan D. H., Johnson W. D., Myers V. H., Prather T. L., McGlone M. M., Rood J., … Sjöstrom L. V. (2010). Nonsurgical weight loss for extreme obesity in primary care settings: Results of the Louisiana obese subjects study. *Archives of Internal Medicine*, 170(2), 146–154. 20101009 10.1001/archinternmed.2009.508

[CIT0045] Sarlio-Lähteenkorva S. (2000). ‘The battle is not over after weight loss’: Stories of successful weight loss maintenance. *Health (London)*, 4(1), 73–88.

[CIT0046] Setchell J. P., Gard M. P., Jones L. P., & Watson B. M. P. (2017). Addressing weight stigma in physiotherapy: Development of a theory-driven approach to (re)thinking weight-related interactions. *Physiotherapy Theory and Practice*, 33(8), 597–610. 28590789 10.1080/09593985.2017.1328718

[CIT0047] Sharma A. M. (2017, 7 13). The key to obesity management lies in the science of energy homeostasis. Retrieved from http://www.drsharma.ca/the-key-to-obesity-management-lies-in-the-science-of-energy-homeostasis

[CIT0048] Stubbs J., Whybrow S., Teixeira P., Blundell J., Lawton C., Westenhoefer J., … Raats M. (2011). Problems in identifying predictors and correlates of weight loss and maintenance: Implications for weight control therapies based on behaviour change. *Obesity Reviews*, 12(9), 688–708. 21535362 10.1111/j.1467-789X.2011.00883.x

[CIT0049] Stuckey H. L., Boan J., Kraschnewski J. L., Miller-Day M., Lehman E. B., & Sciamanna C. N. (2011). Using positive deviance for determining successful weight-control practices. *Qualitative Health Research*, 21(4), 563–579. 20956609 10.1177/1049732310386623 PMC3612888

[CIT0050] Svenaeus F. (2000). The body uncanny - Further steps towards a phenomenology of illness. *Medicine, Health Care and Philosophy*, 3(2), 125–137. 10.1023/a:1009920011164 11079340

[CIT0051] Thomas J. G., Bond D. S., Phelan S., Hill J. O., & Wing R. R. (2014). Weight-loss maintenance for 10 years in the national weight control registry. *American Journal of Preventive Medicine*, 46(1), 17–23. 24355667 10.1016/j.amepre.2013.08.019

[CIT0052] Toombs K. (1993). *The meaning of illness*. Dordrecht, Netherlands: Kluwer Academic Publishers.

[CIT0053] van Manen M. (1997). *Researching lived experience: Human science for an action sensitive pedagogy*. London, ON: The Althouse Press.

[CIT0054] van Manen M. (2014). *Phenomenology of practice*. Walnut Creek, CA: Left Coast Press.

[CIT0055] van Wijngaarden E., van der Meide H., & Dahlberg K. (2017). Researching health care as a meaningful practice: Toward a nondualistic view on evidence for qualitative research. *Qualitative Health Research*, 27(11), 1738–1747. 28799478 10.1177/1049732317711133

[CIT0056] Wing R. R., & Hill J. O. (2001). Successful weight loss maintenance. *Annual Review of Nutrition*, 21, 323–341. 10.1146/annurev.nutr.21.1.323 11375440

[CIT0057] Wing R. R., & Phelan S. (2005). Long-term weight loss maintenance. *American Journal of Clinical Nutrition*, 82(1), 222S–225S. 16002825 10.1093/ajcn/82.1.222S

[CIT0058] World Health Organization (2017a). BMI classification. Retrieved 28 8 2017, from http://apps.who.int/bmi/index.jsp?introPage=intro_3.html

[CIT0059] World Health Organization (2017b). Body mass index - BMI. Retrieved 28 8 2016, from http://www.euro.who.int/en/health-topics/disease-prevention/nutrition/a-healthy-lifestyle/body-mass-index-bmi

[CIT0060] Yumuk V., Tsigos C., Fried M., Schindler K., Busetto L., Micic D., & Toplak H. (2015). European guidelines for obesity management in adults. *Obesity Facts*, 8(6), 402–424. 26641646 10.1159/000442721 PMC5644856

